# Serological study of small ruminant lentiviruses in sheep population of Khorasan-e-Razavi province in Iran

**Published:** 2015-09-15

**Authors:** Behnaz Norouzi, Alireza Taghavi Razavizadeh, Mohammad Azizzadeh, Ashraf Mayameei, Vahid Najar Nezhad Mashhadi

**Affiliations:** 1 *Department of Clinical Sciences, Faculty of Veterinary Medicine, Ferdowsi University of Mashhad, Mashhad, Iran; *; 2 *Department of Pathobiology, Faculty of Veterinary Medicine, Ferdowsi University of Mashhad, Mashhad, Iran; *; 3 *Department of Internal Medicine, Faculty of Veterinary Medicine, Urmia University, Urmia, Iran*

**Keywords:** ELISA, Iran, Khorasan-e-Razavi, Seroprevalence, Small ruminant lentiviruses

## Abstract

Maedi-Visna (MV) virus and caprine arthritis encephalitis (CAE) virus known as small ruminant lentiviruses (SRLVs) cause chronic diseases in susceptible animals. The main reservoirs of these viral agents are sheep and goat. In sheep, MV virus causes a disease as the same name of the virus. This is the first seroprevalence survey of SRLVs in sheep population of Khorasan-e-Razavi province in Iran. Two hundred and twenty sheep from 30 flocks in 12 regions of the province were selected by random cluster sampling method. Serum samples were analyzed for the presence of antibodies against MV/CAE viruses. The seroprevalence in sheep was 34.5% (95.0% CI: 28.3 to 40.7%). Totally, the seroprevalence was in the range of 6.7 to 72.2 %. In 26 flocks of sheep (89.6%; 95.0%CI: 74.4 to 98.8%), at least one seropositive case was detected. The relationship between seropositivity and age, sex, flock size and breeds of sheep were statistically analyzed. In logistic regression model, only age was correlated with SRLV seroprevalence (*p* < 0.05). This study showed relatively high seroprevalence against SRLVs in sheep population in this area of the country. Due to difficulty in clinical diagnosis, chronic course of the disease, the absence of effective vaccine and treatment and huge economic loss, more epidemiological studies with regards to prevention and control of the disease are necessary.

## Introduction

Maedi-Visna virus (MVV) andcaprine arthritis-encephalitis virus (CAEV) are known as small ruminant lentiviruses (SRLVs). Both of them cause chronic diseases in small ruminants. MVV is closely related to CAEV.^1^ Although documented cases of natural cross-species transmission are currently rare, MVV can infect goats and CAEV can infect sheep.^2^

Maedi-Visna (MV) or ovine progressive pneumonia is an economically important viral disease of sheep that occasionally affects goats.^2^ Maedi-Visna is a composite name, originally Icelandic, used to describe two slowly progressive infectious disease of sheep, which share a common viral etiology.^3^ Maedi meaning dyspnea that is a progressive interstitial pneumonia and Visna meaning wasting that refers to meningoencephalitis.^3^

Maedi-Visna is caused by a retrovirus of the subfamily lentiviridae.^4^ The disease was first described in Iceland in 1940 and from that time the disease has been reported in many of the sheep rearing countries of the world except for Australia and New Zealand.^3^

The incubation period of this subclinical infection is usually more than two years and its clinical signs appear when the animal is 3 to 4 years old.^2^ Consuming contaminated colostrum and milk and inhalation of infectious respiratory secretions in close contact are the main routes of transmission.^5^ Generally, both horizontal and vertical transmission has been proposed for MV virus.^4^ After entry of the virus into the body, the host is infected lifelong.^2^

The economic losses of the disease are due to mortality associated with clinical disease, poor value of the removed animals and reduction of economic life.^6^ Effects of subclinical infection on the reproductive potential should also be added to the economic losses.^7^ There is no treatment and effective vaccine against the disease but by increasing the quality and efficiency of diagnostic tests, there is the possibility of eradicating the disease.^8^ Due to the persistence of circulating antibodies against the MV virus, detection is mainly based on serological tests including agar gel immunodiffusion (AGID), enzyme-linked immunosorbent assay (ELISA) and indirect immunofluorescence.^9^

## Materials and Methods

**Area of study**. The study was carried out on sheep population in Khorasan-e-Razavi province. The province is located on the north-east part of Iran, geographically the latitude and longitude of Khorasan-e-Razavi is 35.1020253 and 59.1041758, respectively.^10^ It covers an area of about 144,681 km^2^ consisting of 25 cities and its center is Mashhad. This province have a common border with Turkmenistan (53.6 km) and Afghanistan (302.0 km) with annual mean temperature and precipitation about 14.5 ˚C and 250 mm, respectively.^11^

Considering the seroprevalenceof 2.18% reported by Sakhaee and Khalili for Maedi-Visna virus,^6^ a minimum sample size of 149 sheep was required for detecting disease, where the probability of finding at least one case in the sample is 95.0%.^12^

Blood samples were collected over a period of one year (January 2009 – February 2010) from 220 sheep (178 females and 42 males) in 12 regions of the province including Quchan, Sarakhs, Fariman, Torbat-e-Heydarieh, Torbat-e-Jam, Khaf, Kashmar, Nishabur, Sabzevar, Gonabad, Kalat and Mashhad.

**Sampling**. Sampling was done in each region from 1 to 5 flocks by multi-stage cluster random sampling method. Blood samples were collected in vaccutainer tubes without anticoagulant. All blood samples were centrifuged for 10 min at 3000 rpm and separated sera were stored at – 20 ˚C until analysis. Any hemolysed samples were discarded.

**Enzyme-linked immunosorbent assay test**. Detection of MVV/CAEV antibody in sera was performed using ELISA Maedi-Visna/CAEV antibody test kit (Institut Pourquier, Montpellier, France), based on manufacture instruction. The sensitivity and specificity values for the test were 97.0 and 90.3, respectively. ^13^

The bottom of paired columns of 96-well microplates of the kit, were coated with an immunogen peptide called p28 recombinant protein which is a viral capsid protein. Briefly, each of serum samples, positive and negative controls was diluted to a ratio of 1:20 by the diluent buffer (Phosphate buffered saline/PBS-tween 20; Institut Pourquier, Montpellier, France) and 200 µL of each was placed in each test plate. Plates were incubated at 37 ˚C for 1 hr (± 5 min). After washing and adding 100 µL of diluted (1/100) anti-ruminant IgG HRPO conjugate (Institut Pourquier, Montpellier, France), microplate was incubated for 30 min (± 3 min) and then washing was repeated. Finally, the reaction was revealed with 100 µL of tetramethylbenzidine, then it stopped after 20 min with adding 100 µL of stop solution 3 (0.5 Molar sulphuric acid, Institut Pourquier, Montpellier, France) per well.

Reading of optical density (OD) was performed using microplate reader (ELX800; BioTek, Winooski, USA) at 450 nm. Interpretation of the OD results was performed based on calculation of sample to positive (S/P) ratio for each sample by this formula: 

$S/P=\mathrm{100}\times \frac{\mathrm{OD 450 value of the sample}–\mathrm{OD 450 value of the negative control}}{\mathrm{Mean OD 450 value of the positive control}–\mathrm{OD 450 value of the negative control}}$

Sera were considered positive when the ratio of (S/P) was higher than or equal to 120%. Doubtful results were recorded when the S/P ranged between 110% and 120%. Samples with an S/P ≤ 110% were considered negative.

**Statistical analysis**. Herd and animal level sero-prevalence and a 95% confidence interval were calculated. At the first step, univariate analysis of relationship between each independent variable and seropositivity was performed using the chi-Square test. Predictors with *p*-value < 0.20 were placed into a logistic regression model. A backward stepwise approach was used to identify explanatory variables which are related to the seropositivity. We removed variables that were not significantly correlated with seropositivity from the model one at a time, beginning with the least significant, until the estimated regression coefficients for the retained variables were significant at an alpha level of < 0.05. All statistical analysis performed using SPSS statistical software (Version 16; SPSS Inc., Chicago, USA). Considering the sensitivity and specificity of the test, the real seroprevalence calculated using the following equation:

## Results

Out of 220 heads of sheep, 76 heads were positive for the presence of antibodies against SRLVs. Seroprevalence (apparent seroprevalence) in sheep was 34.5% (95.0% CI: 28.3 - 40.7%). Considering the sensitivity and specificity of the test, the real seroprevalence calculated as 29.6%.

**Fig. 1:**
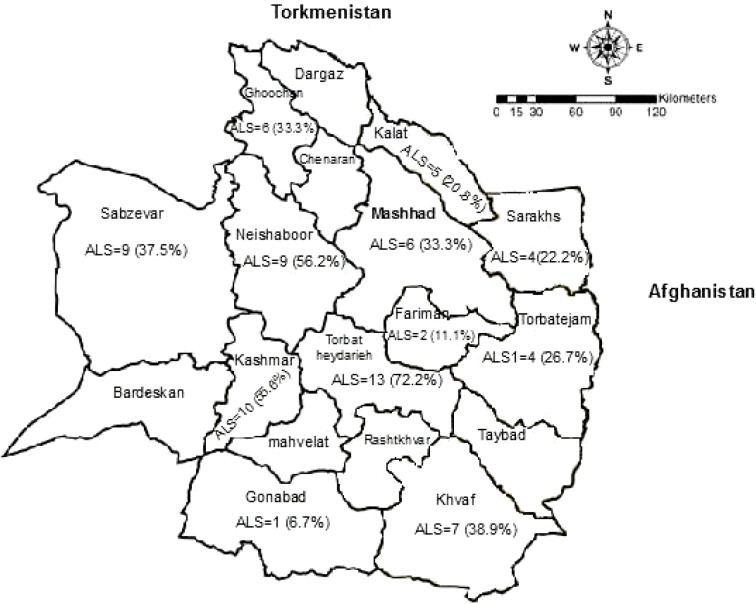
Distribution of positive cases in sheep population of the regions in the Khorasan-e-Razavi. Animal Level Seroprevalence (ALS) number (percentage) of positive animal

Out of 30 studied flocks, at least one positive serum sample was found in 26 (89.6%; 95.0% CI: 74.4 to 98.8%) flocks. Antibodies against SRLVs were detected in all selected regions. The lowest and highest seropositivity rate were in Gonabad (6.7%) and Torbat-e-Heydarieh (72.2%), respectively. Distribution of the infection and status of seroprevalence in studied regions is shown in Fig. 1.

Number and proportion of seropositive animals with respect to different levels of independent variables and the results of univariate analysis showing the association of each independent variable with seroprevalence are shown in Table 1. Age, sampling area and flock size showed a significant relationship with seroprevalence in univariate analysis (*p* < 0.05).

**Table 1:** Proportion of positive cases for different levels of independent variables and the association of each of the independent variables with disease caused by small ruminant lentiviruses in sheep

**Variables **		**No.**	**Positive (%)**		**CI** * ** (95%)**	***p*** **-value**
***Sex***						
	**Female**	178	63(35.4%)	0.34-0.36		0.586
	**Male**	42	13(31.0%)	0.29-0.33		
***Age (year)***						
	**1 - 2**	65	16(24.6%)	0.24-0.26		0.014
	**≥ 2**	111	45(40.5%)	0.40-0.42		
	**< 1**	30	5(16.7%)	0.15-0.19		
***Strain***						
	**Afshari**	30	12(40.0%)	0.37-0.43		0.862
	**Balouchi**	47	17(36.2%)	0.34-0.38		
	**Kordi**	76	24(31.6%)	0.31-0.33		
	**Others**	67	23(34.3%)	0.33-0.35		
***Region***						
	**Fariman**	18	2(11.1%)	0.08-0.15		0.001
	**Khaf**	18	7(38.9%)	0.34-0.44		
	**Sabzevar**	24	9(37.5%)	0.34-0.41		
	**Torbat-e-** **Heydarieh**	18	13(72.2%)	0.67-0.77		
	**Kashmar**	18	10(55.6%)	0.5-0.61		
	**Sarakhs**	18	4(22.2%)	0.18-0.27		
	**Kalat**	24	5(20.8%)	0.18-0.24		
	**Nishabur**	16	9(56.2%)	0.5-0.62		
	**Gonabad**	15	1(6.7%)	0.03-0.1		
	**Torbat-e Jam**	15	4(26.7%)	0.21-0.32		
	**Quchan**	18	6(33.3%)	0.28-0.38		
	**Mashhad**	18	6(33.3%)	0.28-0.38		
***Flock size***						
	**< 300**	37	21(56.8%)	0.54-0.6		0.006
	**300 - 1000**	133	38(28.6%)	0.28-0.3		
	**> 1000**	50	17(34.0%)	0.32-0.36		

* **CI: **Confidence interval

Independent variables which were associated with infection by the Chi-square test (*p* > 0.2), were entered into multivariate logistic regression models. Backward stepwise method was used for selection of variables associated with infection (*p* < 0.05). The results of this test is shown in Table 2. In the final logistic regression model, only age was significantly associated with serum test results.

In logistic model, the ages lower than one year was considered as reference. The chance of seropositivity increased by the age. The likelihood of finding a positive outcome in sheep with the age of more than two years old was 3.4 (95.0% CI: 1.2-9.5) folds greater than sheep with the age of lower than one year old (*p *= 0.02).

**Table 2:** Results of multivariate logistic regression model showing the factor influencing the risk of seropositivity in sheep population

**Variables**	**Levels**	**β** 1	**SE** 2	***p*** **-value**	**OR** 3	**CI** 4 ** (95%)**
	Constant	-1.61	0.49			
**Age (year)**						
	<1	Reference			1.0	
	1-2	0.490	0.568	0.388	1.63	0.54-4.97
	≥2	1.23	0.527	0.020	3.41	1.21-9.57

1 **β: **Regression coefficient;

2 **SE: **Standard error**; **

3 **OR: **Odds ratio;

4 **CI: **Confidence interval.

## Discussion

The results of the present study showed relatively high seroprevalence against SRLVs in sheep population in the north-east of Iran.

A large number of similar studies have been performed in the other parts of the world. The difference in the prevalence of an infectious disease in different regions is evident. For example, in the study by Shuaib *et al*. in Quebec, Canada, the seroprevalencerate have ranged from 7.6 to 59.1%.^8^ In a similar study on 274,048 samples from 544 sheep flock in Aragon in northeastern Spain, the seroprevalence of the disease was determined 52.8%.^14^ Azkur *et al*. in their study on 279 sheep and 146 goats in the Central Anatolia region of Turkey reported the seroprevalence of antibodies against the MVV in sheep and anti-CAEV in goats 19.4 and 7.5%, respectively.^15^ In a survey done by Mahmood *et al*. on sera of 93 sheep and 123 goats by serum AGID in Pakistan, the seroprevalence was reported 7.5 and 3.87%, respectively.^16^

Using AGID, the seroprevalence of the infection in Ethiopia among 250 sheep was reported 70.4% by Seyoum *et al*.^17^

Lamontagne *et al*. using complement fixation test on 708 sera from 182 sheep flocks in 3 areas of ​​Canada reported the seroprevalence rate among 20 to 67.6%.^18^ Giangaspero *et al*. in Japan using three methods AGID, ELISA and polymerase chain reaction (PCR) on serum samples from 267 sheep reported the prevalence 1.1%, 0% and 0% for each method, respectively.^19^

In order to assess the sensitivity and specificity of three tests (AGID, ELISA and PCR), Karanikolaou *et al*. did a study on 143 sheep in infected flocks in Greece and found that ELISA is more sensitive than the other methods.^20^ Thus, three points of fact could be emphasize, first, the kind of diagnostic tests is effective on results, second, ELISA is a rapid, simple, sensitive and specific method for detection of specific antibodies against MVV in ruminants' serum samples and third, to improve the sensitivity and specificity, it is better to use a combination of methods for detection of SRLVs infection.

In the present study, although age, sampling area and flock size had significant relationship with seropositivity in univariate analysis, in multivariate logistic regression model the age of sheep was the only variable which was significantly associated with seroprevalence. It shows that the mean age of sampled sheep might be different in different regions and flocks.The probability of infection in sheep older than two years was significantly higher than the age of less than one year. In this regard, the findings of this study is consistent with Gufler *et al*. and Arsenault *et al*. studies. ^21^^,^^22^

In this study, no significant relationship between herd size and seroprevalence was observed in multivariate analysis while in the study of Huttner *et al*., it was noted that the rate of seroprevalence in large flocks (> 250 heads) is more than smaller flocks (10 - 100 heads)^23^ and it has also been proposed by Arsenault *et al*. ^22^ in Canada and Simard *et al*.^24^ It should be noted that in this study, very little flocks had the size of less than 100 sheep and the size of most flocks were large. Despite the lack of significant effect of herd size on seroprevalence, analysis of results indicated that in the flocks less than 300, the percentage of infection were relatively higher. It seems that differences in management, which is probably due to differences in farmers work experience, and economical aspects are effective.

The difference in the prevalence of an infectious disease in different regions of a country is unavoidable, for example the seroprevalence of Maedi-Visna in different parts of Quebec varied from 14.5% to 69.7%.^8^ Some factors such as different susceptibility of different breeds in studied regions, management practices and the biosecurity affect on the prevalence ofthe disease. Two later factors are also related to weather conditions and experience and economic statue of farmers. It seems that, in regions with cold weather such as Quchan, keeping the sheep in a dense rearing manner may increase the risk of horizontal transmission of the disease. 

Since there is no vaccine or effective treatment against this disease, in countries with low prevalence, the policy of serological diagnosis of the disease and exclusion of the affected animals has been proposed as the control principle. However, in areas with a high prevalence due to heavy financial losses, quarantine policy and the gradual elimination of seropositive animals is recommended. The control of the infection in animal populations will limit economic losses resulting from clinical disease.

It is recommended to find out effective control and prevention measures, similar studies should be performed in the other provinces of Iran, especially the provinces which are located near the neighboring countries. 
